# Differential Adhesive Properties of Sequestered Asexual and Sexual Stages of *Plasmodium falciparum* on Human Endothelial Cells Are Tissue Independent

**DOI:** 10.1371/journal.pone.0031567

**Published:** 2012-02-21

**Authors:** Francesco Silvestrini, Marta Tibúrcio, Lucia Bertuccini, Pietro Alano

**Affiliations:** 1 Dipartimento di Malattie Infettive, Parassitarie e Immunomediate, Istituto Superiore di Sanità, Rome, Italy; 2 Dipartimento Tecnologie e Salute, Istituto Superiore di Sanità, Rome, Italy; Université Pierre et Marie Curie, France

## Abstract

The protozoan parasite *Plasmodium falciparum*, responsible for the most severe form of malaria, is able to sequester from peripheral circulation during infection. The asexual stage parasites sequester by binding to endothelial cell receptors in the microvasculature of various organs. *P. falciparum* gametocytes, the developmental stages responsible for parasite transmission from humans to Anopheles mosquitoes, also spend the almost ten days necessary for their maturation sequestered away from the peripheral circulation before they are released in blood mainstream. In contrast to those of asexual parasites, the mechanisms and cellular interactions responsible for immature gametocyte sequestration are largely unexplored, and controversial evidence has been produced so far on this matter. Here we present a systematic comparison of cell binding properties of asexual stages and immature and mature gametocytes from the reference *P. falciparum* clone 3D7 and from a patient parasite isolate on a panel of human endothelial cells from different tissues. This analysis includes assays on human bone marrow derived endothelial cell lines (HBMEC), as this tissue has been proposed as a major site of gametocyte maturation. Our results clearly demonstrate that cell adhesion of asexual stage parasites is consistently more efficient than that, virtually undetectable of immature gametocytes, irrespectively of the endothelial cell lines used and of parasite genotypes. Importantly, immature gametocytes of both lines tested here do not show a higher binding efficiency compared to asexual stages on bone marrow derived endothelial cells, unlike previously reported in the only study on this issue. This indicates that gametocyte-host interactions in this tissue are unlikely to be mediated by the same adhesion processes to specific endothelial receptors as seen with asexual forms.

## Introduction

*Plasmodium falciparum*-infected erythrocytes are characterized by their ability to adhere to the endothelial cells lining the microvasculature of various organs. Adhesion of the asexual (trophozoite/schizont) stage-infected erythrocytes and their sequestration away from the peripheral circulation is implicated in malaria pathogenesis. *In vitro* binding assays with erythrocytes infected with asexual-stage parasites have revealed specific interactions between one or more receptors on the host endothelium and parasite-encoded ligands on the infected erythrocytes. Host cell receptors CD36 and ICAM-1 (CD54) are thought to be the major receptors in the adhesion of most *P. falciparum* isolates [1]. Members of the *P. falciparum* Erythocyte Membrane Protein-1 (PfEMP-1) family of variable surface-expressed parasite antigens have been shown as parasite ligands mediating adhesion of asexual-stage-infected erythrocytes.

In *P. falciparum* not only asexual stages are able to sequester in internal organs. A portion of parasites in the bloodstream does not progress into the asexual cycle but differentiate into gametocytes, the parasite stages able to mature into gametes when engorged in the blood meal of a biting Anopheles mosquito, and therefore responsible of Plasmodium transmission from humans to the insect vector. *P. falciparum* gametocytes mature in about ten days, in an approximately five time longer period than asexual stages, in which they undergo morphological transformations classically divided in five distinct stages [2]. Only gametocytes at the last developmental stage (V) are normally detectable in peripheral blood of infected individuals. Immature gametocytes (stages I to IV), like asexual stages, have instead the ability to sequester in poorly defined body sites, from which they are released only when they reach maturity.

In contrast to the above described studies on asexual forms, the adhesion of erythrocytes infected with sexual-stage parasites has been poorly described. After early reports from the first years of malariology describing bone marrow and spleen as the organs where all stages of gametocyte maturation are readily found, followed by few recent confirmations [3]–[5], systematic studies on sites of gametocyte sequestration are still not available. The only information currently available on gametocyte cytoadhesion is contained in a few reports using cell lines, on which binding of stages II to V gametocytes, stages clearly recognizable by morphology, was measured. Gametocyte adhesion has been explored by Rogers *et al.* [6] in a static-binding assay on C32 amelanotic cell line, which constitutively express both ICAM-1 and CD36, reporting that mid-stage gametocytes (stage III–IV) are able to adhere to such cells, although at a lower level compared to asexual stages. In this study asexual and gametocyte adhesion was inhibited to a similar extent by anti-CD36 and anti-ICAM-1 antibodies, which led authors to suggest a common role of these receptors in mediating cytoadhesion. This conclusion was however not confirmed by the work of Day et al. [7], where adhesion phenotypes of different gametocyte stages of the parasite clone 3D7 (and of 6 additional isolates and clones) were assessed in binding assays on the above cell line and on purified ICAM-1 and CD36. No adhesion of mid-stage gametocytes was observed in that work either on C32 cells nor on purified ligands. In another report specifically investigating the cellular interactions possibly governing gametocyte sequestration in bone marrow, Rogers *et al.* [8] used for the first time endothelial cell lines derived from human bone marrow (HBM) endothelium and stroma to compare binding of 3D7 asexual and mid- and mature sexual stages. Results were that mid-stage gametocytes showed a low binding affinity to these cell lines, which was nevertheless comparatively higher than that observed for asexual parasites. The main ligand involved in such binding, based on antibody inhibition experiments, was proposed to be ICAM-1. Stage V gametocytes did not show any appreciable binding, consistent with their condition of being freely circulating cells.

In order to re-assess the controversial conclusions of the above reports, the present work provides a systematic comparison of asexual and sexual stage cell binding properties conducted on a panel of endothelial cell lines derived from different tissues and expressing different levels of the main ligands - ICAM-1 and CD36 - proposed above to be implicated in gametocyte binding. Cell lines used were commercially available primary lines HUVEC and HDMEC and two HBM-derived cell lines, HBMEC-60 and HBMEC-33 [9], used here for the first time in malaria research. The latter lines were developed and are currently used to study the cellular and molecular interactions between hematopoietic progenitor cells (HPC) and bone marrow endothelium responsible for homing, endothelial transmigration and interplay governing transplantation efficiency of hematopoietic precursors [10]–[12]. Analysis of baseline and induced expression of 44 surface molecules and receptors along several weeks of cultivation showed that such lines stably maintain the specific features of primary BM derived endothelial cells. In order to directly compare results with the above reports on gametocyte adhesion, most of the experiments were similarly carried out with the stable gametocyte producer parasite clone 3D7. Besides high gametocyte production, this clone stably retains the ability to adhere to C32 melanoma cells without selection by panning on host cells or ligands [6], [13]. Part of this remarkable phenotypic stability is probably due to the fact that long term propagation of this parasite clone is not accompanied by major chromosomal rearrangements [13], particularly subtelomeric deletions responsible for loss of knob production, cytoadhesion and gametocytogenesis [14]. Finally, in order to provide a dataset directly comparable to a variety of parasite cell binding studies in *P. falciparum*, adhesion experiments in this work were performed conforming to protocols currently used in studying *P. falciparum* asexual stage cytoadherence [15].

## Results

**Figure 1:**
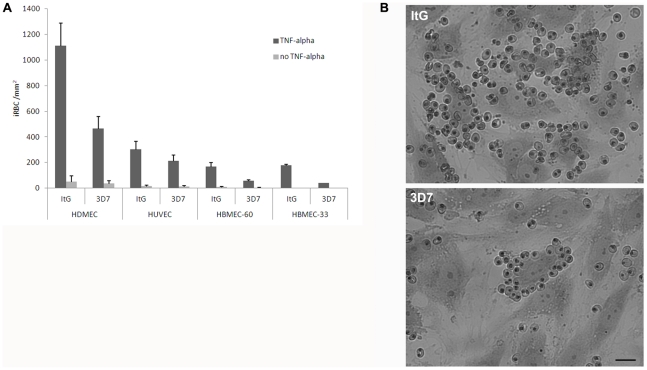
Adhesion of asexual stages of ItG and 3D7 on stimulated and non stimulated HDMEC, HUVEC and HBMEC endothelial cell lines. A) Data shown are the mean number of iRBC per mm^2^ ± S.E. of 3 to 5 biological replicates. Static assays were carried out as described in the Material and Methods section. B) Giemsa-stained infected erythrocytes bound to TNF-alpha activated endothelial cells (HDMEC). Scale bar: 25 µm.

In order to ensure comparability of the present experiments with state of the art cytoadhesion studies in *P. falciparum* [15], cell binding assays were performed using parasites directly taken from culture, rather than after Percoll or gelatin/plasmagel purification. Appropriate volumes of uninfected blood were used to adjust parasite numbers and culture hematocrit in comparing different parasite stages/samples. Two preliminary sets of experiments were performed with the HUVEC, HDMEC and HBMEC-60 endothelial cell lines. In the first, basal expression and TNF-alpha mediated upregulation of CD36 and ICAM-1 were measured with specific antibodies in the three endothelial cell lines. This experiment (Figure S1) confirmed that the host ligands were induced by the cytokine, and showed that HUVEC and HBMEC are essentially ICAM-1-positive/CD36-negative, while HDMEC are ICAM-1/CD36-double positive [9], [16], thus providing cues to the functional role of such receptors in this comparative analysis. In the second asexual parasite binding efficiencies in presence/absence of TNF-alpha were measured. In these experiments the *P. falciparum* clone ItG, a reference clone in cytoadhesion studies whose stable cytoadherent phenotype is maintained by panning selection on HDMEC cells [17], and the gametocyte producer clone 3D7 were used. Endothelial cells were grown to confluence, and exposed to TNF-alpha (0.5 ng ml−1) for 12 h or left untreated. Equal numbers of late trophozoites from synchronous asexual cultures of ItG and 3D7 were adjusted to 1% hematocrit, and incubated for 2 h. After removal of unbound uninfected and infected erythrocytes, cell monolayers were fixed and stained by Giemsa, and the numbers of bound parasites per mm^2^ of cell layer were counted. Results of experiments (Figure 1) confirmed that TNF-alpha is a potent inducer of the host ligands mediating asexual parasite adhesion, and suggested to undertake the subsequent gametocyte adhesion assays in TNF-alpha–stimulated cells. These experiments also showed that 3D7 asexual infected erythrocytes maintain a stable cytoadherent phenotype not only on C32 melanoma cells as mentioned above [13] but also on the panel of endothelial cells. Data on binding of 3D7 parasites to endothelial cells are scarce in the literature despite this being a reference clone in malaria research, and this experiment provides, to our knowledge, the first systematic comparison of adhesion of asexual stages of this clone. The comparison shows that 3D7 has a generally lower binding efficiency than ItG, which could be partly explained by the fact that 3D7 parasites used here were not routinely selected by panning. Another likely explanation is that 3D7 cytoadhesion is efficiently mediated by CD36, which is poorly expressed by HUVEC and HBMEC and is more abundantly produced on HDMEC cells, whilst it is reported that ItG binding relies on both CD36 and ICAM-1, and the latter being efficiently stimulated by TNF-alpha on the surface of all the above endothelial cells [17].

### Cytoadhesion of 3D7 asexual and sexual stages

**Figure 2:**
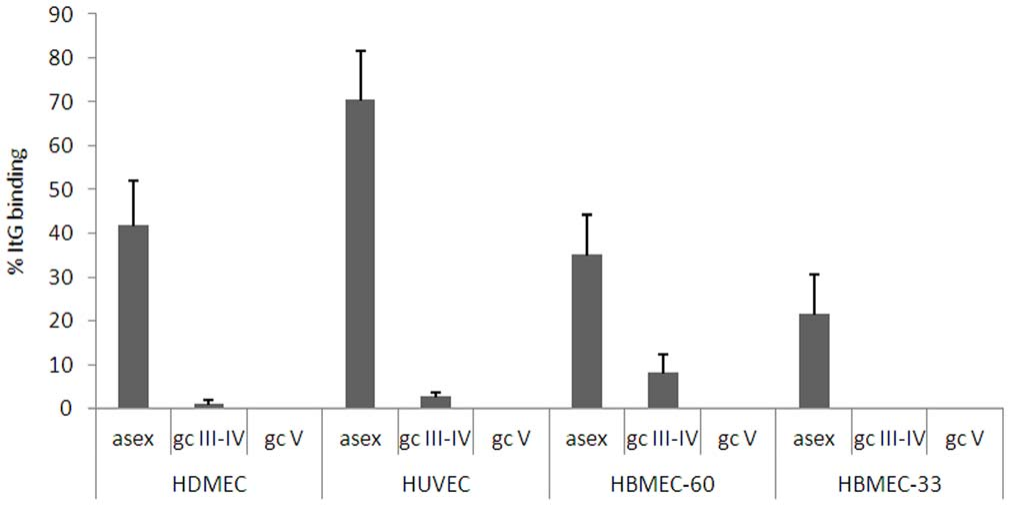
Adhesion of asexual and sexual stages of 3D7 on TNF-alpha stimulated HDMEC, HUVEC and HBMEC cell lines. Static assays were carried out as described in the Material and methods section. The mean number of iRBC per mm^2^ ± S.E. of 3 to 5 biological replicates were counted, and are expressed as % of bound parasites per mm^2^ of the ItG control.

Having confirmed the requirement of TNF to upregulate expression of the relevant host ligands, a comparative analysis of adhesive properties of asexual parasites and immature (stage III–IV) and mature (stage V) gametocytes was conducted on the above panel of endothelial cell lines. Experiments always included ItG asexual parasites as positive control, enabling expression of cell binding efficiencies of 3D7 asexual and sexual stages as percent of ItG cell binding (Figure 2). Unfortunately, the failure of the ItG clone to produce gametocytes prevented the possibility to directly compare asexual and sexual stage binding in this genetic background. One result of these experiments clearly showed that mature stage V gametocytes fail to bind to HUVEC, HDMEC and the newly used HBMEC lines at any measurable levels. This extends previous observations that mature gametocytes do not bind to C32 melanoma cells and the HBM endothelial and stromal cell lines used in [8] to the panel of endothelial cell lines used here. The most relevant result of this analysis is however that mid-stage gametocytes show a significantly lower adhesion to endothelial cells from all tissues/organs tested compared to asexual parasites, and that importantly this applies also to the bone marrow derived HBMEC cells. This observation is in contrast to that reported by the only available study addressing this issue [8], and does not support the hypothesis that specific host-gametocyte adhesive interactions are responsible for sequestration of immature sexual stages in bone marrow.

### Parasite adhesion to endothelial cells in presence of Interleukin-1 beta and bone marrow Stromal-Derived Factor-1

**Figure 3:**
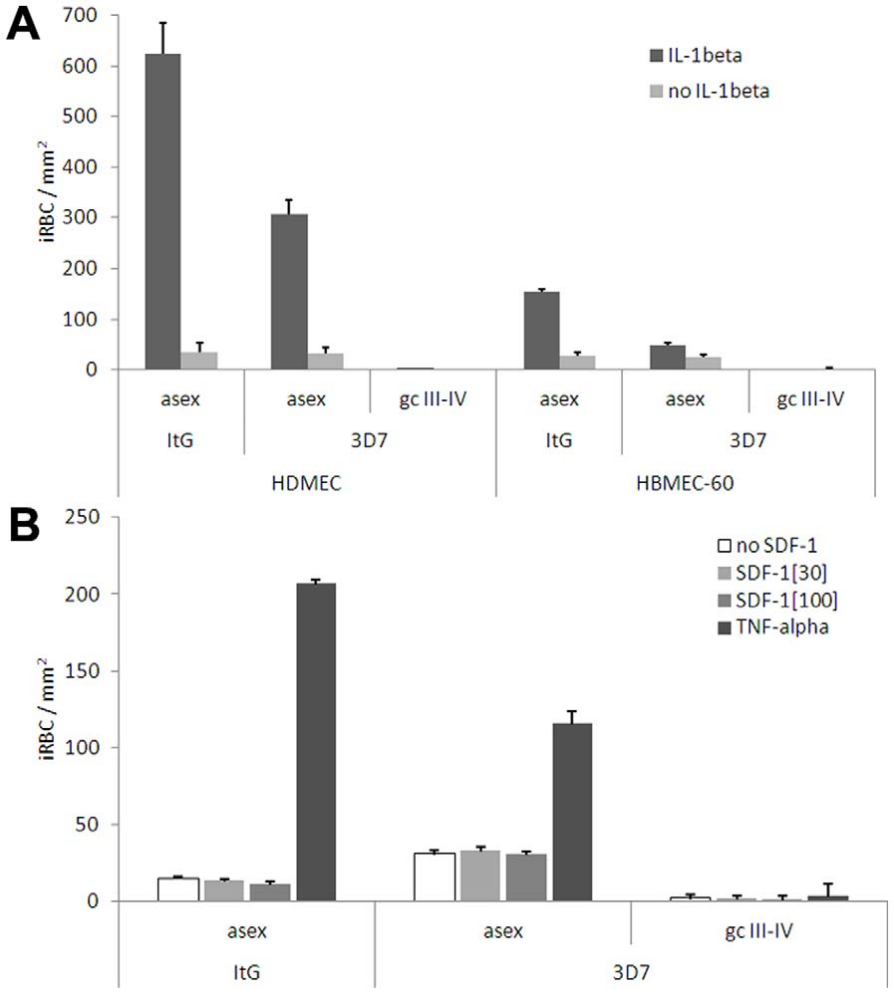
Influence of IL-1beta and SDF-1 on adhesion of asexual and sexual stages of 3D7 on endothelial cells. A) Adhesion of asexual and sexual stages of 3D7 on HDMEC and HBMEC-60 stimulated with IL-1beta. B) Adhesion of asexual and sexual stages of 3D7 on HBMEC-60 with different concentrations of SDF-1. Static assays were carried out as described in the Material and methods section. Data shown are the mean number of iRBC per mm^2^ ± S.E. of 2 to 5 biological replicates.

In order to further investigate asexual stage and gametocyte binding to endothelial cells, the possible role of additional signaling molecules beside the inflammatory cytokine TNF-alpha was investigated. One experiment measured effect of Interleukin-1beta (IL-1beta), another inflammatory cytokine mediating host ligand upregulation [18], [19], on the binding of asexual parasites and mid-stage gametocytes. HDMEC and HBMEC cells were exposed to 10 U per ml of IL-1beta for 6 h, and adhesion assays were performed as described above. Results show that, although IL-1beta was able to stimulate asexual stage binding on HDMEC and HBMEC endothelial cells, as observed for TNF-alpha, no difference could be observed for gametocyte binding, which was virtually undetectable as observed in the same cells stimulated by TNF-alpha (Figure 3 A). In another experiment, binding of the same parasite stages was measured in response to increasing concentrations of Stromal-Derived Factor-1 (SDF-1). This cytokine plays a role in bone marrow physiology as the major chemoattractant for homing, adhesion and extravasation of hematopoietic progenitor cells (HPC) [20]. For this reason only HBMEC cells were used in this experiment. Such cells were incubated with 0, 30 and 100 ng per ml of SDF-1 for 2 h, and asexual and sexual stage binding performed as above. Result of this experiment (Figure 3 B) showed that, in the conditions tested, SDF-1 had no effect in promoting adhesion of mid-gametocytes. In addition the experiment showed that, unlike observed with the above inflammatory cytokines, SDF-1 did not promote an increased binding of asexual stages.

### Adhesion of sexual and asexual stages of a gametocyte-producing parasite isolate

**Figure 4:**
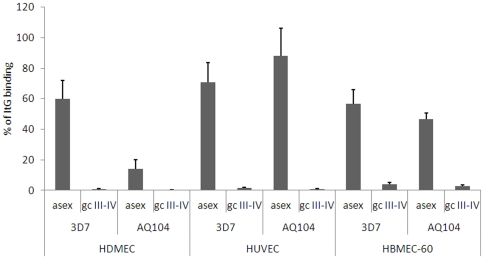
Adhesion of asexual and sexual stages of AQ104 and 3D7 on TNF-alpha stimulated HDMEC, HUVEC and HBMEC cell lines. Static assays were carried out as described in the Material and methods section. Values are expressed as % of bound parasites per mm^2^ of the ItG control. Data shown are the mean number of iRBC per mm^2^ ± S.E. of at least 3 biological replicates was counted and expressed as % of bound parasites per mm^2^ of the ItG control.

The parasite clone 3D7 is able to successfully propagate through mosquitoes, and its use in human volunteer experimental infections indicates that this parasite is fully competent to infect humans [21], [22]. In order to nevertheless rule out that the observed failure of 3D7 gametocytes to appreciably bind to host cells was due to an unrecognized deficiency due to laboratory adaptation, a set of adhesion assays on the panel of endothelial cells was also performed with gametocytes from a recently, independently adapted parasite isolate with an unrelated genetic background. The African isolate AQ104 was obtained in the field study described in [23] and was observed to produce gametocytes for a few weeks of cultivation (Dr. S. Borrmann, University of Heidelberg, personal communication). It was thus possible to use freshly thawed parasite cultures of AQ104 to induce sexual differentiation and obtain mid-stage gametocytes for this experiment. Adhesion assays were performed on HDMEC, HUVEC and HBMEC with asexual and mid-stage gametocytes from AQ104, in parallel with asexual and sexual stages from clones 3D7 and ItG asexual parasites. Results of these experiments showed that asexual stages of AQ104 were able to adhere to endothelial cells of the different lines with efficiencies comparable to those shown by 3D7 (Figure 4). Importantly, the binding of mid-gametocytes produced by AQ104 was invariably significantly lower than observed for asexual stages of the same line, and was hardly detectable as observed for the sexual stages produced by 3D7. This confirmed that, in the conditions of this experiment, mid-stage gametocytes from a genetically distinct isolate from 3D7 also exhibit minimal, if any, cytoadherence efficiency.

## Discussion

Mechanisms of cytoadherence of asexual stages of *P. falciparum* have been extensively studied and different *in vitro* models to measure static and flow adhesion of these parasites to endothelial cells have been developed [24]. On the contrary, the hypothesis that sexual stages may interact through specific molecules with the human endothelium is still controversial, partly because several experiments measured gametocyte adhesion on non-endothelial cell lines [6], [7]. The present work aimed to further address this issue by systematically comparing adhesion of asexual and sexual stages of *P. falciparum* on a panel of endothelial cell lines from various human organs/tissues. We dedicated special attention to specific aspects of the binding assay protocol. The first was to avoid artifacts in cell adhesion possibly introduced by parasite enrichment procedures using colloids such as Percoll [25]. We noticed that Percoll gradient purification of asexual or sexual stages was sometimes associated to detection of higher numbers of bound parasites of both stages compared to untreated parasite cultures. On the other hand use of magnetic columns (MACS) [26] for enrichment of pigmented asexual or sexual stage parasites often resulted in abundant presence of free haemozoin granules in the binding assays, particularly in the case of sexually induced cultures which need to reach high asexual parasitaemias. Free pigment was hardly removed by washing steps and was thought to possibly interfere with binding and with accurate parasite counts on the endothelial cell monolayers. For the above reasons and, importantly, to conform to routine protocols used to measure asexual stage adhesion, all cell binding assays were here performed by simply diluting cultures with parasites of the desired stages, and identifying the bound asexual and sexual cells by their typical morphologies. Other features of the experiments were to consistently expose the endothelial cell monolayers to equal numbers of parasites in all samples, and to routinely include in all binding assays the reference internal positive control of the ItG clone to confidently compare different experiments.

A major result of the present comparative analysis is that immature, mid-stage gametocytes, equivalent to those able to sequester in natural infections, consistently show a dramatically lower cell binding efficiency than isogenic asexual parasites on the entire panel of endothelial cell lines. An obvious difference between asexual stages and the immature gametocytes analyzed in this study (stage III–IV) is that the sexual stages do not modify the surface of the infected red blood cells with knobs [27], the cellular structures where the major parasite ligand PfEMP1 is exposed [14]. It should be however noticed that static binding assays such as those used in this study are able to detect the binding, albeit at lower efficiencies, of asexual parasites lacking knobs but still expressing low levels of PfEMP1 [14]. Failure to detect gametocyte binding in the present assays therefore suggests that such a ligand, or alternative ligands with similar specificity, are likely to be absent from the surface of the red blood cells infected by stage III–IV gametocytes.

In this study asexual stage parasites showed different cell binding efficiencies, varying between parasite clones and endothelial cell lines used, whilst the poor binding values observed for sexual stages were independent from the endothelial cell lines used. Importantly, immature gametocytes from both the reference laboratory clone 3D7 and from a newly established parasite isolate do not exhibit a comparatively higher binding efficiency to HBM-derived endothelial cell lines compared to lines from other host tissues. This result does not support a previous hypothesis suggesting that sequestration of sexual stages in the bone marrow is possibly mediated by interactions between specific ligand on the host cells and the sexual stage parasites [8]. Although the question of the role of bone marrow as a major maturation site for *P. falciparum* gametocytes is awaiting a state of the art re-examination, several reports indeed stated that developing gametocytes are readily found in post mortem analyses and in aspirates from this tissue [3], [28]. In this respect it is interesting to mention that specific human cell types such as haematopoietic precursor cells (HPCs) and metastatic cells from some epithelial tumors, also find in bone marrow, a preferential homing/maturation site [29]. However, when HPCs were tested in cell adhesion assays on endothelial cell lines of different origin, they failed to show a higher binding efficiency on endothelial cells of HBM origin, suggesting that additional factors are needed to promote such specific interactions [9]. In the case of HPCs and of several of the above tumor cells, a major role as a chemoattractant is played by SDF-1 [30], [31]. A potential role of this factor in triggering the binding of gametocytes to HBM endothelial cells was tested in the present work. In this case, binding assays were performed in absence of TNF-alpha or IL-beta stimulation to mimic the non-inflammatory condition of bone marrow in a typical non symptomatic gametocyte carriers [32]. No effects of SDF-1 were however observed in promoting adhesion of gametocytes, and of asexual parasites, in the experimental conditions used here. An additional consideration of the possible host-gametocyte interactions in the HBM environment is that blood circulation in this tissue occurs through sinusoids in which the discontinuous endothelium structure physiologically allows cells to pass in and out of circulation [33]. It is therefore conceivable that gametocytes have direct access to stromal cells with the absence of significant slow flow in this environment. In such a case gametocytes would not need to have asexual stage-like properties largely mediated by ICAM-1 and CD36 for being retained in this environment, and/or could rely on different classes of ligands unrelated to those typically exposed on endothelial cells.

Cell binding assays cannot recapitulate the cellular and topological complexity displayed by human tissues, and that this caveat particularly applies to intricate microenvironments such as the bone marrow niche. The present work nevertheless confirms that asexual stages expose parasite surface molecules able to efficiently bind host ligands and to mediate adhesion like in their natural sequestration sites, and shows that in contrast gametocyte-infected red blood cells fail by the same criteria to appreciably show such a phenotype. The consequent hypothesis that minimal levels, if any, of parasite ligands are present on gametocyte-infected erythrocytes is in agreement with the recent observations that no specific antigens could be identified on the surface of immature gametocytes [34]. This on one hand may be consistent with the requirement of the developing gametocyte to keep a very low antigenic profile in the long period of its sequestered maturation. On the other hand this suggests that specific high affinity interactions between host and parasite encoded/induced ligands are not used by immature gametocytes as the major mechanism of parasite sequestration, in contrast to what is known for asexual stages. Speculation on additional, or alternative, mechanisms used by immature gametocytes to maintain sequestration may be suggested by recent observations that alteration of mechanical properties of erythrocytes infected by asexual parasites can affect the spleen retention process [35] and that red blood cells infected by immature gametocytes are significantly more rigid than those infected by mature gametocytes (M. Tibúrcio, personal communication).

Elucidation of the mechanisms of gametocyte sequestration and identification of the involved body sites are obviously still awaiting definitive answers. The renewed interest in such fundamental issues of *P. falciparum* biology is however promising that understanding and interfering with such mechanisms will hopefully soon become an additional avenue in the effort to eliminate malaria.

## Materials and Methods

### Parasite culture

*P. falciparum* clones 3D7A [36], ItG [17] and isolate AQ104 were grown in 0+ red blood cells in RPMI 1640 plus hypoxanthine 50 mg/ml, supplemented with 10% heat-inactivated 0+ human naturally-clotted serum, at 37°C, in a 2% O_2_ and 5% CO_2_ atmosphere. The cryo-preserved isolate AQ104 [23] was thawed by a stepwise replacement of glycerol with salt (NaCl) and systematically adapted to culture conditions. Parasites were cultured in complete cell culture medium (RPMI 1640 supplemented with L-glutamine, 0.1 mM hypoxanthine, gentamicin, 2% heat-inactivated AB serum, and 5% Albumax II) in the presence of 0+ or A+ blood at 5% hematocrit and a gas mixture of 5% CO_2_, 5% O_2_ and 90% N_2_. The parasite population was kept between 0.1 and 10% (parasites/erythrocytes) with regular change of medium and the addition of fresh blood.

For parasite synchronisation, cultures at 8–10% parasitaemia at 10% haematocrit were centrifuged at 3000 rpm for 10 min through a 60% Percoll cushion and slow sedimenting schizonts used to reinvade fresh red blood cells. Prior to use, parasites were washed twice in binding buffer (RPMI 1640 medium, supplemented with 6 mM glucose, pH 7.2) and re-suspended at 1% haematocrit and 3% parasitaemia (Giemsa staining). Induction of gametocytogenesis was performed as follows; 2% synchronous trophozoites were incubated overnight on a shaker to obtain around 10% ring stage parasites on day 1. On day 2 the culture was split to obtain a schizont parasitaemia of approximately 2%. About 40 h after reinvasion from merozoites produced by such schizonts stage I gametocytes were observable in culture amongst unhealthy asexual parasites. The latter were cleared by incubation with N-Acetylglucosamine for 48 h to obtain virtually pure gametocyte cultures, which were maintained with daily change of medium until they reached the required maturation stages.

### Endothelial cells

Characterised human umbilical vein endothelial cells (HUVEC) and human dermal microvascular endothelial cells (HDMEC) were obtained from PromoCell (Heidelberg, Germany), while human bone marrow endothelial lines, HBMEC-60 and 33 [9], were kindly provided by Dr. E van der Schoot (CLB, Sanquin Blood Supply Foundation, The Netherlands). HUVEC and HDMEC cells were routinely grown in gelatin-coated culture flasks and in Endothelial Cell Grown Medium (PromoCell, Heidelberg, Germany) supplemented with 10% (v/v) heat-inactivated FCS, while for HBMEC cells Lonza BulletKit® medium (Lonza AG, Switzerland) was used.

### Flow Cytometry

The expression of endothelial cell markers was measured by fluorescence-activated cell sorting (FACS). Specific fluorescence-conjugated antibodies were used: APC Mouse anti-human CD54 (ICAM-1) and PE Mouse anti-human CD36 (Becton Dickinson, CA). Nonspecific fluorescence was assessed using corresponding isotype control antibodies. After 16 hours of cytokine activation, endothelial cells culture medium was gently removed and cells were washed once with fresh medium, followed by three washes with phosphate-buffered saline (PBS). Endothelial cells were detached by gentle trypsin (Sigma, UK) treatment, washed with PBS containing 1% BSA and stained with antibodies for 60 min on ice in 100 ml of the recommended dilution. Cells were washed twice in PBS with 1% BSA and a FACS analysis was carried out using a FACSCanto (Becton Dickinson, CA). The expression of surface molecules was indicated by geometric mean of the fluorescence intensity.

### Static cell assay

Static cell binding assays were carried out using a modified version of a previously described method [14]. Endothelial cells (6th passage) were seeded onto 1% gelatin coated 13 mm Thermanox coverslips (Nalgene, Nunc). Once confluent, they were incubated overnight at 37°C with or without 0.5 ng ml−1 rTNF-alpha or rIL-1beta (Invitrogen UK). Cells were washed with binding buffer supplemented with 1% (v/v) of naturally-clotted human serum and incubated with 0.5 ml of parasite suspension (3% parasitaemia, 1% haematocrit) for 1 h at 37°C, with gentle resuspension every 10 min. After washes in binding medium, coverslips were placed upside down in new clean microplate wells filled with fresh binding medium for 30 minutes. The remaining unbound cells detached from the endothelial layer sinking down by gravity. The procedure was repeated twice. After removal of unbound cells, adherent cells were fixed using 1% glutaraldehyde for 1 h and then stained with 5% Giemsa for 30 min. For cytokine stimulation, recombinant human SDF-1 alpha (CXCL12, PeproTech Germany) was added at different concentrations to the parasites suspension during cells incubation for 2 hours at 37°C. Coverslips were dried and mounted on slides using DPX mounting buffer (BDH Lab Supplies). For each condition/line bound parasites in triplicate wells were counted at 400× magnification and their numbers expressed as the number of infected RBC per mm^2^. All experiments were repeated at least three times.

## Supporting Information

Figure S1 Constitutive and TNF-alpha stimulated expression of endothelial cells markers ICAM-1 and CD36 in HUVEC, HDMEC and HBMEC-60 cell lines. Expression levels were determined by FACS and expressed as geometric means of fluorescence intensity and percentage of positive cells.
(PDF)
