# Supplementary material for: Differential Adhesive Properties of Sequestered Asexual and Sexual Stages of Plasmodium falciparum on Human Endothelial Cells Are Tissue Independent
Source: PLoS One. 2012 Feb 21;7(2):e31567. doi: 10.1371/journal.pone.0031567 (PMC3283655; doi:10.1371/journal.pone.0031567)

ICAM-1 (CD54)

Geometric Mean of Fluorescence

	constitutive	pos.cells (%)	TNF stimulated	pos.cells (%)
**HUVEC**	44.73	7.99	261.91	84.34
**HDMEC**	93.68	15.36	126.74	72.59
**HBMEC-60**	48.11	3.96	238.12	96.65

CD36

Geometric Mean of Fluorescence

	constitutive	pos.cells (%)	TNF stimulated	pos.cells (%)
**HUVEC**	32.56	1.08	35.81	10.37
**HDMEC**	251.53	33.35	480.65	45.59
**HBMEC-60**	49.6	4.84	53.32	20.66

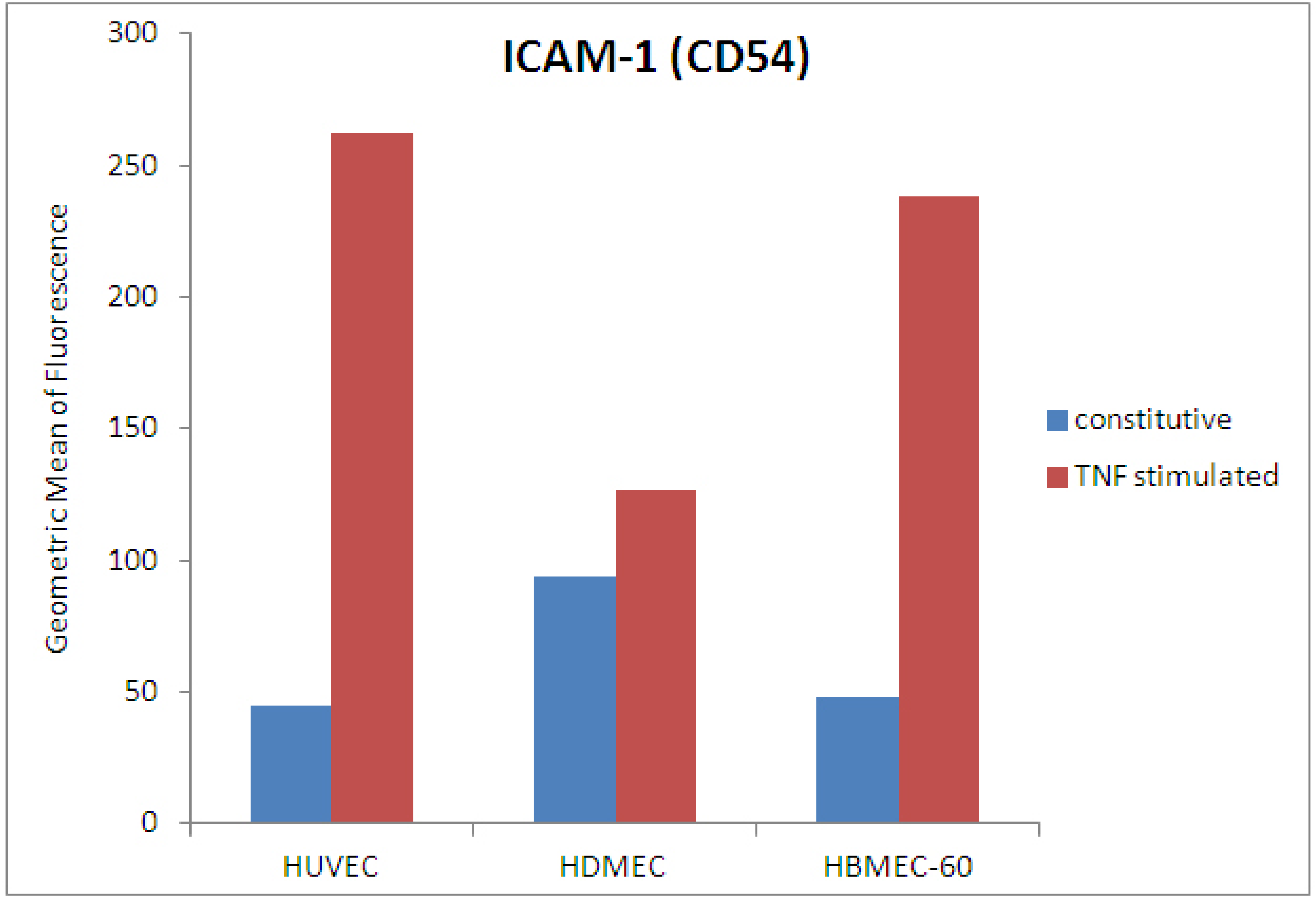

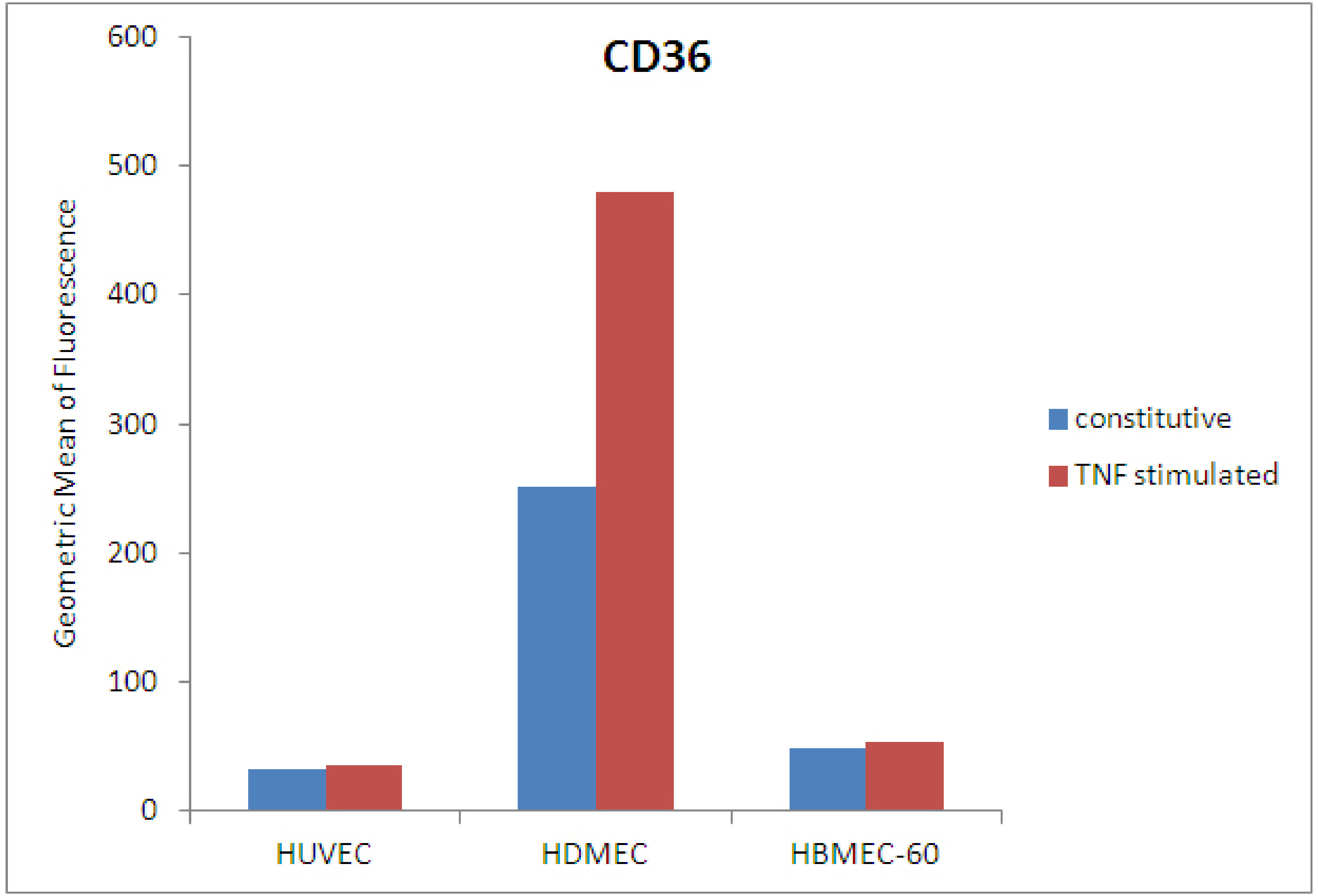

Supplement: Figure S1 — Constitutive and TNF-alpha stimulated expression of endothelial cells markers ICAM-1 and CD36 in HUVEC, HDMEC and HBMEC-60 cell lines. Expression levels were determined by FACS and expressed as geometric means of fluorescence intensity and percentage of positive cells. (PDF) [file pone.0031567.s001.pdf]
